# Body Mass Index, Waist Circumference, Body Fat, Fasting Blood Glucose in a Sample of Moroccan Adolescents Aged 11–17 Years

**DOI:** 10.1155/2012/510458

**Published:** 2011-11-28

**Authors:** Slimane Mehdad, Abdeslam Hamrani, Khalid El Kari, Asmaa El Hamdouchi, Amina Barakat, Mohamed El Mzibri, Najat Mokhtar, Hassan Aguenaou

**Affiliations:** ^1^Unité Mixte de Recherche en Nutrition et Alimentation, (URAC 39), Centre National de l'Energie, des Sciences et Techniques Nucléaires, Université Ibn Tofaïl, Kenitra, Morocco; ^2^Equipe de Recherche en Santé et Nutrition du Couple Mère-Nouveau-Né, Faculty of Medicine and Pharmacy, Rabat 10001, Morocco

## Abstract

*Objectives*. The study aimed to assess the relationship between body fat and each of body mass index (BMI) and waist circumference (WC), and to test the effectiveness of fat mass (FM), percent of body fat (PBF), BMI, and WC in predicting high levels of fasting blood glucose (FBG). *Methods*. A total of 167 adolescents aged 11–17 years were recruited from Rabat region. BMI and WC were determined using standard equipments. FM and PBF were derived from isotope dilution technique. FBG was determined by the hexokinase method. *Results*. Regardless of the weight status, BMI showed a strong positive correlation with FM and PBF in both genders. WC was significantly correlated with FM in boys and girls, and with PBF in different groups of girls and boys of the study sample. However, there was no significant relationship between WC and PBF in normal weight and overweight-obese groups of boys. FBG was highly correlated with FM and PBF in girls of the study sample and in overweight-obese girls. Similar significant relationship between FBG and both BMI and WC was observed in overweight-obese girls, while there was no significant association between FBG and other variables in boys and normal-weight girls. *Conclusion*. BMI and WC were closely associated with FM and PBF, respectively. However, the degree of these associations depends on gender and weight status. BMI may provide a better proxy estimate of overall adiposity than WC; nevertheless, both of them would appear to be a reasonable surrogate for FM and PBF as screening tools to identify adolescents at risk of developing excess body fat and high level of FBG.

## 1. Introduction

Over the last decades, there has been a worldwide growing prevalence of overweight and obesity among people of all ages [1, 2]. Obesity and its incidence reached epidemic levels and have become major public health concerns [3]. The real risk factor of obesity is an excess in adiposity which is strongly associated with adverse health outcomes, including diabetes mellitus, dyslipidemia [4], blood pressure [5] coronary disease, kidney disease, cancer, musculosketal consequences, asthma, and decreased fertility [6]. 

Given the major short- and long-term consequences of childhood obesity on health, well-being, and costs to health care and social security systems, as well as the better chances for intervention at young ages, public and private funding agencies should give a high priority to research on obesity in children and adolescents [7]. Early identification of adolescents at risk for excess adiposity and its related metabolic complications requires reliable, simple, and specific measures of excess body fat for this age group [8]. 

To this end, a set of techniques are used to assess obesity such as isotope dilution and dual energy X-ray absorptiometry (DEXA). These techniques offer accurate measurement of adiposity but they are expensive and cannot be used everywhere [9]. Thus, measuring body fat in most clinical and epidemiological settings is relatively difficult, and surrogate anthropometric measures, such as body mass index (BMI; kg/m^2^) and waist circumference (WC) are used for assessment of obesity in children and adolescents.

A number of studies have explored the relationship between BMI and adiposity measures and showed high degree of correlation between them in children and adolescents [9–12]. However, BMI provides misleading information about body fat [13, 14] and its clinical interpretation remains controversial [15]. Other studies have reported strong positive correlation between WC and body fat [16, 17], and WC has been advocated as an indicator of central obesity [18, 19]. However, it was indicated that WC may overestimate total and trunk fat [20] and it is not clear which of the central adiposity measures best predict the overall adiposity [21].

The goals of this study were to assess, for the first time in Morocco and North Africa: (a) the relationship between body fat, assessed by isotope dilution technique, and each of BMI and WC in adolescents, and (b) the effectiveness of fat mass (FM), percent body fat (PBF), BMI, and WC to predict adolescents with high-blood glucose level as health risk related to excess body fat.

## 2. Materials and Methods

### 2.1. Study Design and Data Collection

The study was carried out in Rabat region (Morocco) after receiving the institutional approval from the Ministry of National Education. A total of 167 adolescents (123 girls and 44 boys) aged 11–17 years were recruited from seven randomly selected secondary schools. The adolescents who participated in the study were selected by their teacher based on their weight status (overweight/obese and normal weight). A written consent was obtained from the parents or tutors, and verbal consent was provided by each subject. Anthropometric measures, saliva, and blood samples were taken at schools. Fat mass, percent body fat and fasting blood glucose level were determined at the laboratory of “Unité Mixte de Recherche en Nutrition et Alimentation, URAC39 (Université Ibn Tofaïl-Centre National de l'Energie, des Sciences et Techniques Nucléaires-CNESTEN-Rabat).”

### 2.2. Anthropometry

Anthropometric measurements were taken by trained operators using standard equipments. Body weight was measured to the nearest 0.1 Kg using portable scale (Seca, Germany) with minimal clothing and no shoes. Height was measured to the nearest 0.1 cm using a height bar (2 meters, dismantling) without shoes. BMI was calculated as weight in kilogram divided by the square of height in meter (Kg/m^2^). WC was measured to the nearest 0.1 cm in standing position at the midpoint between the lowest rib and the iliac crest and at the end of normal expiration, using a measuring tape.

Using these measurements and the new WHO growth reference 5–19 years [22], the weight status of each subject was categorized: obese (z-score > +2SD, equivalent to BMI > 30 kg/m² at 19 years), overweight (z-scores > +1SD, equivalent to BMI > 25 kg/m² at 19 years), and normal weight (−2SD ≤ z-scores ≤ +1SD, equivalent to 18 ≤ 25 kg/m² < BMI ≤ 25 kg/m² at 19 years).

### 2.3. Body Composition Determined by Isotope Dilution Technique (Deuterium Oxide)

In our study, FM and PBF were estimated from total body water (TBW). TBW was determined by isotope dilution technique using the deuterium oxide (^2^H_2_O). Naturally, the body water pool contains a small amount of deuterium (^2^H). This represents the natural abundance of ^2^H in body water. When ^2^H is ingested, it mixes with body water within a few hours. The amount of deuterium in body water above that naturally present is known as the enrichment of body water that reaches a “plateau” after 3–5 hours [23]. Each adolescent received orally a dose of ^2^H_2_O (0.5 g/kg body weight). The saliva samples were taken at baseline, after an overnight fast, and at 3 h after ingesting the ^2^H (endpoint). The level of ^2^H in saliva samples was measured by Fourier transform infrared spectroscopy (FTIR) [24]. TBW was calculated from the saliva sample by the plateau method, assuming that this plateau was reached at 3 hours [25]. The following equations were used [23]:

(1) Deuterium  space(L)=Dose  amount  (mg)(Enrichment  H2  in  saliva (mg/kg)).

In order to correct the in vivo isotope exchange in calculating TBW, it is necessary to divide by 1.041 [26]

(2) $$\text{TBW}\;\left(\text{L}\right)=\frac{\text{Deuterium}\;\text{space}\;\left(\text{L}\right)}{1.041}.$$

FM and PBF were calculated from TBW using the following equations:

(3) $$\begin{array}{c} \text{Fat}\;\text{free}\;\text{mass}\;\left(\text{FFM}\right)\;\left(\text{kg}\right)=\frac{\text{TBW}\;\left(\text{L}\right)}{\text{Hydration}\;\text{factor}},\\ \text{FM}\;\left(\text{kg}\right)=\text{Weight}\;\left(\text{kg}\right)-\text{FFM}\;\left(\text{kg}\right),\\ \text{PBF}\;\left(\text{\%}\right)=\frac{\text{FM}\;\left(\text{kg}\right)}{\text{Weight}\;\left(\text{kg}\right)}\times 100. \end{array}$$

In this study, we used the hydration factors (see Table 1) for children and adolescents [27].

### 2.4. Fasting Blood Glucose

All subjects had fasted for 12 hours prior to blood draw. Blood samples were stored in ice till the delivery to the laboratory (within 4 hours), and subsequently stored at −80°C until analysis. Fasting blood glucose (FBG) concentration was measured using the glucose hexokinase methodology [28].

### 2.5. Statistical Analysis

Means and standard deviations were calculated for each variable using descriptive statistics. Two-way ANOVA was used to examine the effect of gender, weight status, and their interaction. Pearson's correlation was used to assess the relationship between body fat (FM and PBF) and each of BMI and WC, and their association with FBG. All statistical analyses were performed using SPSS (statistical package for social sciences, version 17.0). The Kolmogorov-Smirnov normality test was used to determine whether data set was well modeled by a normal distribution or not. *P* values < 0.05 were considered significant.

## 3. Results

A total of 167 adolescents participated in the study. 42% were overweight or obese and 58% had normal weight. Since the number of obese and overweight subjects was low, boys and girls of the study sample were divided into two groups (normal weight and overweight-obese). The mean and standard deviations (SD) of age, weight, height, BMI, WC, FM, PBF, and FBG are presented in Table 2. Statistical analyses showed that PBF was significantly higher in girls than boys (*P* = 0.001), while there was no gender effect on the other variables. Weight, BMI, WC, FM, and PBF were significantly higher in overweight-obese groups compared to normal weight groups (*P* < 0.0001); however weight status had no effect on FBG. Regarding the interaction between gender and weight status, it had a significant effect on BMI (*P* = 0.025), while it had no effect on WC, FM, PBF, and FBG.

Table 3 shows the correlations between body fat and each of BMI and WC. BMI was positively correlated to FM, with Pearson's correlation coefficients (*r*) above 0.57, in both genders of the study sample (boys, *r* = 0.850; girls, *r* = 0.896: all *P* < 0.0001) and in different weight status groups (boys: normal weight, *r* = 0.770; overweight-obese, *r* = 0.739; girls: normal weight, *r* = 0.690; overweight-obese, *r* = 0.799: all *P* < 0.0001). BMI was also significantly correlated with PBF in both genders of the study sample (boys, *r* = 0.711; girls, *r* = 0.724: all *P* < 0.0001) and in normal weight boys (*r* = 0.648, *P* = 0.005), overweight-obese boys (*r* = 0.413, *P* = 0.032), normal weight girls (*r* = 0.505, *P* < 0.0001), and overweight-obese girls (*r* = 0.488, *P* = 0.001). 

On the other hand, WC showed significant positive correlation with FM in both genders of the study sample (boys, *r* = 0.717; girls, *r* = 0.824: all *P* < 0.0001) and in different weight status groups (boys: normal weight, *r* = 0.513, *P* = 0.035; overweight-obese, *r* = 0.571, *P* = 0.002; girls: normal weight, *r* = 0.626, *P* < 0.0001; overweight-obese, *r* = 0.628, *P* < 0.0001). Similarly, significant correlation between WC and PBF was seen in both genders of the study sample (boys, *r* = 0.575; girls, *r* = 0.677: all *P* < 0.0001), in normal weight and overweight-obese girls (*r* = 0.434, *P* < 0.0001; *r* = 0.404, *P* = 0.008, resp.). However, there was no significant correlation between PBF and WC in normal weight and overweight-obese boys.

Overall, the relationships of BMI and WC with each of FM and PBF were found to be dependent on gender and weight status. The relationship between BMI and PBF was stronger in overweight-obese girls than overweight-obese boys. Similarly, the relationship between WC and PBF was stronger in both normal weight and overweight-obese girls than normal weight and overweight-obese boys. On the other hand, the association of BMI and WC with FM was observed to be more significant than with PBF mainly in overweight-obese boys.

Table 4 shows the Pearson's correlation coefficients of FBG with FM, PBF, BMI, and WC. FBG was found to be strongly correlated with FM in girls of the study sample (*r* = 0.241, *P* = 0.007) and in overweight-obese girls (*r* = 0.583, *P* < 0.0001). Similar positive correlation was observed between FBG and PBF in girls of the study sample (*r* = 0.246, *P* = 0.006) and in overweight-obese girls (*r* = 0.561, *P* < 0.0001). In addition, a significant correlation was found between FBG and both BMI and WC in overweight-obese girls (*r* = 0.330, *P* = 0.033 and *r* = 0.528, *P* = 0.004, resp.). However, the relationship of FBG to FM, PBF, BMI and WC was not significant in boys. On the other hand, there was a trend toward a negative correlation between FBG and FM, in overweight-obese group of boys, and both BMI and WC in normal weight girls, but these correlations were not significant.

## 4. Discussion

### 4.1. Relationship between BMI and Each of FM and PBF

BMI is commonly used as an indicator of overall obesity in adults due to its simplicity and correlation with percent body fat [29], but its use in children and adolescents is still a controversial issue because it seems to give a limited insight of excess body fat degree [8, 30]. Children with the same BMI may show a noticeable variation in total body fat [13]. Unlike adults, annual increase in BMI during childhood is generally attributed to the lean rather than to the fat component of BMI [31–33]. The association between BMI and PBF in young subjects differs among ethnic groups, and BMI does not fully explain differences in PBF [34].

Our results showed a high significant relationship between BMI and each of FM and PBF in both boys and girls. These results are in agreement with previous studies, suggesting that BMI is highly related to adiposity and may be useful in identifying excess body fat in children and adolescents [35–38] and that correlation of BMI with FM is greater than with PBF [9, 38], we have now confirmed this in a different ethnic sample of adolescents from North Africa. In addition, and most of all, our findings confirm the results of previous studies, indicating the high positive relationship between BMI and PBF among adolescents [11], the role of BMI as a predictor of PBF, and the gender differences in the relationship between PBF and BMI [39].

### 4.2. Relationship between WC and Each of FM and PBF

A number of studies have reported the strong positive correlation between WC and body fat [16, 17]. WC rather than BMI agrees with perception of body size, possibly due to its relation with abdominal fat at different ages [40], and could serve better than BMI and skin fold thickness for identifying central adiposity [41]. WC has been shown to have a significant role in identifying overweight and obese individuals [42]. However, it was indicated that WC may overestimate total and trunk fat [5, 20] and that the relationship between WC and body fat could be influenced by weight status and gender in youth [10]. The present study explored the relationship between WC and each of FM and PBF.

WC was found to be closely associated to FM and PBF in both boys and girls of the study sample, and in normal weight and overweight-obese girls. Our results are consistent with those of previous studies which suggest WC as a good diagnostic test for fatness in adolescents [17, 20]. Regarding the effect of weight status on the relationship between WC and adiposity measures, our results differ in some respects from earlier studies, probably relating to the low number of overweight and obese adolescents who participated in the study. However, these results confirm that the relationship between WC and direct measures of overall adiposity may be influenced by gender. This gender difference was apparent in our study as the correlations were stronger in girls compared to those observed in boys. These finding agree with a previous study which showed that girls have a higher FM than boys and WC may not reflect total fat [5].

### 4.3. Relationship Between FBG and Each of BMI, WC, FM, and PBF

The current study aimed to test the effectiveness of FM, PBF, BMI, and WC in predicting high levels of FBG as health risk related to excess body fat in adolescents. Many studies support the hypothesis that the relationship between adiposity and risk of disease begins early in life [43, 44]. The increased intra-abdominal adipose tissue is the most clinically relevant type of body fat that is associated with metabolic complications and adverse health effects including hyperinsulinemia and type 2 diabetes in childhood [45, 46]. However, it is not yet clear whether this association can be found in youth of all ethnic groups. 

Our results showed high positive association of FBG with FM and PBF in girls of the study sample and in overweight-obese girls as well. Similar positive association of FBG with BMI and WC was observed in overweight-obese girls. Our findings are in line with available data from previous studies on the relationship between adiposity and blood glucose level. It has been reported that the level of FBG was found to be higher in overweight and obese children compared to the normal children [47] and that adolescents with high levels of overall and abdominal adiposities had the least favorable glucose levels [48]. Independently of the amount of fat mass, intra-abdominal fat accumulation was found to be strongly related to insulin resistance and hyperglycemia in obese adolescents [49]. Moreover, it was indicated that overweight and obesity were associated with increased risk for developing Type 2 Diabetes [50–52]. The high significant relationship of FBG to BMI, WC, FM and PBF seen in the current study, especially in overweight and obese girls, may be due to the decreased insulin sensitivity which was found to be strongly associated with excess body fat in previous studies [53–56], while weight loss was found to be associated with a decrease in insulin concentration and an increase in insulin sensitivity in adolescents [57, 58]. Also such positive relationship in overweight-obese group of girls may be explained by the clustering of metabolic syndrome factors which place individuals at risk for Type 2 diabetes as it has been reported in another study [59]. 

On the other hand, there was no significant correlation between FBG and BMI, WC, FM, and PBF in boys, may be due to the small sample size. 

Our study had some limitations such as the small size of the whole sample and weight status groups particularly for boys. Our ability to recruit more subjects was hampered by the surge of influenza A/H1N1 during the course of the study. Also the authorization to access to schools has not been renewed by the concerned authorities for 2010-2011 academic year. 

Another limitation is that the relationship between adiposity measures and each of BMI and WC, in one hand, and between FBG and other variables, on the other hand, may depend on pubertal stages (PS) that were not addressed in our study. For instance, it was observed in a previous study that the relationship between WC and PBF changes with sexual maturity, and that the normal pattern from PS1 to PS5 is for PBF to decrease and WC to increase [60].

## 5. Conclusion

BMI and WC were closely associated with FM and PBF, derived from isotope dilution technique, in a sample of Moroccan adolescents from Rabat. It should be noted, however, that these associations depend on gender and weight status, and that BMI may provide a better proxy estimate of overall obesity than WC. Nevertheless, both of them appear to be reasonable surrogate for FM and PBF, particularly in epidemiological studies, as screening tools to identify adolescents at increased risk of developing excess body fat and high levels of fasting blood glucose.

Further research is needed for this group of population and should include (1) studies to confirm our results taking into account the puberty stage, (2) investigation of the association of overall and central obesity with fasting blood glucose level among girls in different age or ethnic groups and the mechanism that produces the gender difference observed in the current study, and (3) relationship of BMI, WC, FM, and PBF with other metabolic abnormalities for early prevention of health risks related to overweight and obesity.

## Figures and Tables

**Table 1:** Hydration factor of fat-free mass in children and adolescents.

Age (years)	Boys	Girls
11-12	75.4	76.6
13-14	74.7	75.5
15-16	74.2	75.0
17–20	73.8	74.5

**Table 2:** Characteristics of study sample (mean ± SD) and main effect of gender, weight status, and their interaction (two-way ANOVA).

Variables	Gender	Total	Normal weight	Overweight-obese		*P *values	
		Boys: *n* = 44 Girls: *n* = 123	Boys: *n* = 17 Girls: *n* = 80	Boys: *n* = 27 Girls: *n* = 43	Effect of gender	Effect of weight status	Effect of interaction between gender and weight status
Age, years	Boys	14.19 ± 0.93	14.44 ± 0.83	14.03 ± 0.96	0.559	0.997	0.041
	Girls	14.29 ± 1.18	14.15 ± 1.15	14.57 ± 1.21			
Weight, kg	Boys	61.64 ± 13.23	55.0 ± 10.0	65.80 ± 13.49	0.185	<0.0001	0.005
	Girls	54.45 ± 13.28	47.4 ± 7.3	67.93 ± 11.52			
Height, m	Boys	1.62 ± 0.11	1.65 ± 0.11	1.60 ± 0.11	0.012	0.284	0.019
	Girls	1.58 ± 0.07	1.58 ± 0.08	1.60 ± 0.06			
BMI, kg/m²	Boys	23.49 ± 3.96	20.12 ± 2.27	25.61 ± 3.27	0.893	<0.0001	0.025
	Girls	21.62 ± 4.47	19.02 ± 2.07	26.58 ± 3.49			
WC, cm	Boys	81.27 ± 11.98	72.4 ± 8.4	86.83 ± 10.52	0.472	<0.0001	0.222
	Girls	75.38 ± 11.76	69.3 ± 7.7	86.98 ± 9.08			
FM, kg	Boys	18.71 ± 10.48	10.64 ± 5.92	23.79 ± 9.53	0.054	<0.0001	0.840
	Girls	17.41 ± 8.90	12.87 ± 4.67	26.07 ± 8.66			
PBF, %	Boys	29.27 ± 12.46	19.08 ± 9.92	35.68 ± 9.26	0.001	<0.0001	0.073
	Girls	30.48 ± 8.92	26.63 ± 6.59	37.83 ± 8.18			
FBG, g/L	Boys	0.92 ± 0.14	0.89 ± 0.11	0.94 ± 0.15	0.306	0.182	0.195
	Girls	0.89 ± 0.11	0.89 ± 0.10	0.89 ± 0.12			

BMI: body mass index (kg/m²); WC: waist circumference (cm): FM, fat mass (kg): PBF, percent body fat (%): FBG: fasting blood glucose (g/L).

**Table 3:** Pearson's correlation coefficients (*r*) of BMI and WC with each of FM and PBF.

	BMI		WC	
	FM	PBF	FM	PBF
Boys				
Total (*n* = 44)	0.850**	0.711**	0.717**	0.575**
Normal weight (*n* = 17)	0.770**	0.648**	0.513*	0.347
Overweight-obese (*n* = 27)	0.739**	0.413*	0.571**	0.289
Girls				
Total (*n* = 123)	0.896**	0.724**	0.824**	0.677**
Normal weight (*n* = 80)	0.690**	0.505**	0.626**	0.434**
Overweight-obese (*n* = 43)	0.799**	0.488**	0.628**	0.404**

*Correlation is significant at *P* < 0.05.

**Correlation is significant at *P* < 0.01.

**Table 4:** Pearson's correlation coefficients of FBG with FM, PBF, BMI, and WC.

	FBG			
	FM	PBF	BMI	WC
Boys				
Total (*n* = 44)	0.121	0.155	0.193	0.214
Normal weight (*n* = 17)	0.180	0.103	0.273	0.214
Overweight-obese (*n* = 27)	−0.049	0.005	0.026	0.270
Girls				
Total (*n* = 123)	0.241**	0.246**	0.094	0.090
Normal weight (*n* = 80)	0.076	0.105	−0.036	−0.142
Overweight-obese (*n* = 43)	0.583**	0.561**	0.330*	0.528**

*Correlation is significant at *P* < 0.05.

**Correlation is significant at *P* < 0.01.

Dependent variable: FBG.

## Abbreviations

BMI: Body mass index
CNESTEN: Centre National de l'Energie, des Sciences et Techniques Nucléaires
FBG: Fasting blood glucose
FFM: Fat-free mass
FM: Fat mass
FTIR: Fourier transform infrared spectroscopy
PBF: Percent body fat
TBW: Total body water
WC: Waist circumference
WHO: World Health Organization.
